# Feline adipose-derived mesenchymal stem cells induce effector phenotype and enhance cytolytic function of CD8+ T cells

**DOI:** 10.1186/s13287-021-02558-5

**Published:** 2021-09-06

**Authors:** Nopmanee Taechangam, Naomi J. Walker, Dori L. Borjesson

**Affiliations:** 1 Department of Pathology, Microbiology and Immunology, University of California, Vet Med 3A, 1285 Veterinary Medicine Mall, Davis, CA 95616 USA; 2 Veterinary Institute for Regenerative Cures, School of Veterinary Medicine, University of California, Davis, CA 95616 USA

**Keywords:** Feline mesenchymal stem cells, Immunomodulation, CD8+ T cells, Granzyme B, Terminally differentiated effector cells

## Abstract

**Background:**

Feline adipose-derived mesenchymal stem cells (ASCs) engage with a variety of immune cells and have been used in several clinical trials for the treatment of inflammatory and immune-dysregulated diseases in cats, but the impact they exert on the functional characteristics on T cells, particularly CD8+ T cells, remains to be elucidated.

**Methods:**

Modified mixed leukocyte reaction was performed between feline ASCs and PBMCs. Changes of cell cycle stages, phenotype and cellular senescence were determined through flow cytometry and gene expression analysis. Cytotoxicity assay was performed to evaluate CD8+ T cell effector function.

**Results:**

Feline ASCs induce cell cycle arrest on CD8+ T cells in a contact-dependent manner, downregulate CD8 surface expression, and shift their phenotype toward terminally differentiated effector cells (CD57+, CD45R+, CD62L−). CD8 T cells interacted with feline ASCs also upregulated granzyme B, IL-2 and KLRG-1 expression and have enhanced cytotoxic potential, evident by the increased percentage of lysis on target cells.

**Conclusions:**

Our findings suggest that feline ASCs (1) alter CD8+ T cells toward terminally differentiated, proinflammatory effector phenotype with limited proliferative capacity, and (2) enhance their cytotoxic potential through granzyme B upregulation. These cytotoxic CD8+ T cells could aid in disease cure in cases caused by an underlying, unresolved viral infection.

## Introduction

Feline adipose-derived mesenchymal stem cells (ASCs) engage with a variety of immune cells and have been used in several clinical trials for the treatment of inflammatory and immune-dysregulated diseases with varying degrees of success [1–4]. However, the mechanisms by which feline ASCs can sustainably alter the immune response remain to be elucidated.

Feline ASCs have effectively treated cats with feline chronic gingivostomatitis (FCGS) [1, 2]. FCGS is a painful, debilitating oral disease with severe inflammation of the gingival tissue. The disease is difficult to successfully manage with standard medical or surgical interventions [5]. Although the cause is multifactorial, FCGS is characterized largely by a persistent inflammatory response from inappropriate T cell activation and tissue infiltration, predominantly with B cells and CD8+ T cells [6]. Underlying viral infection (or antigenic stimulation), particularly secondary to feline calicivirus (FCV), has been implicated [7, 8]. The outcomes of feline ASC interaction with CD8+ T cells have not been explored.

T cell fate decisions are regulated by environmental signals, including growth factors, cytokines, and cell–cell contact. ASCs can regulate T cells through secretion of soluble factors and through direct ligand cell–cell contact. We previously identified a primary soluble mediator, PGE_2_, and a critical ligand interaction, ICAM-1/LFA-1 as necessary for feline ASC inhibition of T cell proliferation via the induction of G0-G1 cell cycle arrest in vitro [9]. Based on the withdrawal of T cells from cell cycle progression, feline ASCs may induce cellular senescence or terminal differentiation.

Our study demonstrated that feline ASCs induce cell cycle arrest in CD8+ T cells in a contact dependent mechanism, alter CD8+ T cell phenotype to terminally differentiated effector cells, and augment their cytotoxic function through upregulation of granzyme B.

## Materials and methods

### Adipose-derived mesenchymal stem cell collection and culture

Adipose-derived feline mesenchymal stem cells (ASCs) were isolated from subcutaneous fat surgically obtained from specific pathogen-free (SPF) cats or from client-owned cats undergoing routine surgery. All owners of client-owned cats signed an informed consent form and conducted with approval of the Institutional Animal Care and Use Committee, University of California, Davis. All cats were free of feline immune deficiency virus (FIV) and feline leukemia virus (FeLV) infection. ASC isolation and expansion was performed as previously described [10]. Briefly, cryopreserved ASCs were thawed in a 37 °C waterbath, seeded into tissue culture flasks with Dulbecco’s modified Eagle’s medium (DMEM low glucose; GIBCO, Grand Island, NY), 10% fetal bovine serum (FBS, Atlanta Biologicals, Flowery Branch, GA), and 1% penicillin/streptomycin (GIBCO) and incubated at 37 °C in 5% CO_2_ at 90% humidity.

Feline ASC lines were characterized by their surface protein expression using flow cytometry. All ASC lines passed quality control assays including bacterial culture (all were sterile), high viability (> 90%), positive for CD90 (identity marker), negative for CD18 (purity marker), and negative for endotoxin and Mycoplasma. All antibodies were purchased from the Leukocyte Antigen Biology Laboratory, University of California, Davis (UCD), unless otherwise indicated. Antibodies included MHC II (42.3), CD18 (FE3.9F2), CD90 (CA1.4G8), CD44 (IM7; BioLegend), and CD105 (SN6; eBioscience). For unconjugated antibodies, a mouse IgG-phycoerythrin antibody (Jackson ImmunoResearch Laboratories) was used for secondary labeling. Canine CD8a (CA9.JD3), rat immunoglobulin G-allophycocyanin (IgG-APC) (eBR2a; eBioscience), and mouse IgG-APC (MCA928; AbD Serotec) were used as isotype controls. Feline ASC lines were obtained from a total of 6 animals between the age of 4.5–11 years from 3 spayed females and 3 castrated males. Low passage (P2-P5) ASCs were used for experiments.

### Feline ASC-PBMC co-cultures

**Fig. 1:**
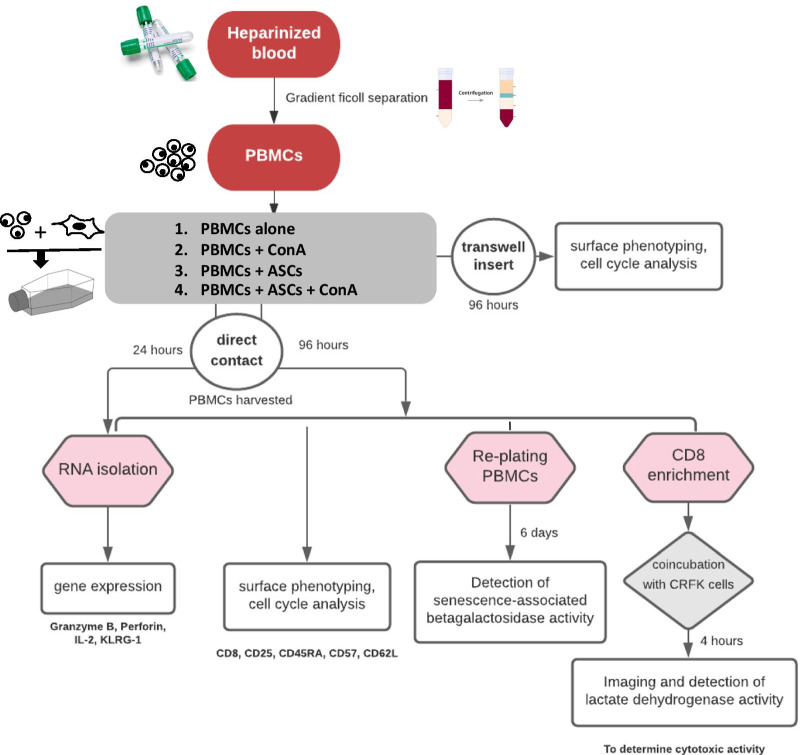
Study design. Image presents the experimental design and timepoints performed. PBMCs were typically harvested at 96 h except for an additional 24 h timepoint for gene expression study. *PBMCs* peripheral blood mononuclear cells, *CRFK cell* Crandell-Reese feline kidney cells, *ConA* Concanavalin A

Feline ASCs were co-incubated with freshly enriched peripheral blood mononuclear cells (PBMCs) with and without the addition of the mitogen ConcavalinA (ConA, 5 µg/mL Sigma-Aldrich, St. Louis, MO) at 1:10 ratio between ASCs and PBMC, as previously described [2]. PBMCs were obtained from 12 healthy colony cats between the age of 2–8 years used for nutritional study at UC Davis, approved by Institutional Animal Care and Use Committee. Four conditions of co-cultures were performed for each experiments in duplicates; PBMCs alone, PBMCs with ConA, PBMCs with ASCs without ConA and PBMCs with ASCs with ConA. Changes were assessed at 96 h, except for gene expression studies with additional 24 h. Experimental design after performing co-cultures is outlined in Fig. 1.

### T-lymphocyte phenotyping

PBMCs were harvested at 96 h after co-cultures and resuspended at a concentration of 1 × 10^6^ cell/mL in flow buffer (DPBS, 1% normal equine serum, 5 mM EDTA and 0.1% sodium azide). Cells were incubated with antibodies for 30 min at room temperature. Antibodies included: mouse anti-feline CD8-PE (clone Fe1.10E9, Leukocyte Antigen Biology Laboratory, UC Davis)—1 μl, anti-feline CD25-FIT C (clone 9F23, a gift from K. Ohno, Tokyo, Japan)—0.2 μl, human anti-mouse CD45R/B220—APC-Cy7 (clone RA3-6B, BioLegend, San Diego, CA)—1.25 μl, human anti-mouse CD57—FIT C (clone TB01, ThermoFisher, Waltham, MA)—1 μl, and human anti-mouse CD62L-APC (clone LAM1-116, MyBiosource.com)—2.5 μl. Reactivity of anti-mouse antibodies against feline T cells were determined by previously reported studies. Multicolor flow cytometry was performed, and cells were analyzed with a Beckman-Coulter Cytomics FC500 flow cytometer with CXP software (Hialeah, FL. Data were analyzed on FlowJo flow cytometry software (Becton Dickinson, San Jose, CA). Cells were pre-gated on CD8+ before further analysis, and single-color beads were used for compensation.

### Cell cycle analysis

Cell cycle analysis was performed as previously described [9]. In brief, PBMCs were incubated with both 7-amino-actinomycin D (7-AAD; BD Biosciences, San Jose, CA), to determine DNA content, and with BrdU, to determine proliferation (FITC BrdU Flow Kit; BD Biosciences) and to distinguish between S-phase, G1 phase and G2-M phase. PBMCs were collected at day 4 from co-incubation experiments with feline ASCs with and without transwell insert. Cells were analyzed with a Beckman-Coulter Cytomics FC500. Data analyses were done on FlowJo flow cytometry software.

### CD8+ T cell enrichment

PBMCs were harvested from the co-cultures at various time-points according to experimental set-up and resuspended in DPBS (Fig. 1). Cells were filtered through a 35-µm cell strainer cap (BD Biosciences), washed (400×*g*, 5 min), and resuspended in MACs buffer (DPBS (GIBCO), 0.5% bovine serum albumin (Fisher Scientific, Santa Clara, CA), 2 mM EDTA (Sigma)). Cells were labeled for CD8 followed by anti-mouse IgG Microbeads (Miltenyi Biotec (San Diego, CA)) and run through a MACS MS separation column (Miltenyi Biotec) per manufacturer's recommendation to obtain a positively selected CD8+ T cell population.

### Senescence associated-β galactosidase (SA-β-Gal) activity

Detection of SA-β-Gal activity was performed with CellEvent™ Senescence Green Flow Cytometry Assay Kit (ThermoFisher Scientific) according to manufacturer’s instruction. Briefly, PBMCs incubated in a standard co-cultures for 4 days and then transferred to a secondary culture for another 6 days before were harvested on day 10, surface stained with anti-CD8 antibodies in flow buffer for 30 min, fixed in 2% paraformaldehyde for 10 min, and incubated with X-Gal substrate for 2 h at 37 °C in the absence of CO_2_. The labeled cells were then analyzed on a flow cytometer (Cytomics FC500) using a 488-nm laser.

### Cytolytic assay: lactate dehydrogenase (LDH) enzyme release assay

Crandell-Reese feline kidney cells (CRFK; target cells) were plated in a 96-well tissue culture plate overnight (phenol-red free Dulbecco’s modified Eagle media (DMEM) and 5% FBS to minimize background interference for colorimetric assay) and infected with Feline Calcivirus (FCV, Kaos strain, generously provided by Dr. Patty Pesavento) at MOI = 0.01. The MOI and infection kinetics of FCV were pre-determined by titrating for an infectious dose that did not create cytopathic effects within the timeframe of the assay. FCV was allowed to infect CRFK cells for 1.5 h, and then isolated CD8+ T cells were co-incubated with the infected target cells for 4 h at 2:1 ratio. CD8+ T cells were co-incubated for four days with ASCs (“primed”) and without ASCs in the presence and absence of activation prior to target cell interaction. After 4 h, the supernatant was collected and LDH activity was determined with CyQUANT™ LDH Cytotoxicity Assay Kit (Invitrogen, Carlsbad, CA) per manufacturer’s instructions and read on a microplate reader at 490 nm and 680 nm (Spectra Max 340; Molecular Devices, San Jose, CA). The cytotoxicity assay was modified according to Weidmann et al [11].

### Gene expression

PBMCs or enriched CD8+ T cells were preserved in RLT buffer and RNA was extracted (RNeasy mini kit, Qiagen, Germantown, MD) and stored at − 80 °C until cDNA was synthesized (High-Capacity cDNA Reverse Transcription Kits, Applied Biosystems, Foster City, CA) per manufacturers’ instructions and stored at − 20 °C until analysis. Quantitative PCR (qPCR) was performed using Power SBYR green (Applied Biosystems) on a QuantStudio 3 Real-Time PCR system (Thermo Fisher). KLRG-1 primers were designed using Primer 3 Plus [12] with sequences from Genebank. Feline perforin, Granzyme B, and IL-2 primer sequences were previously published [13, 14]. Primer information is provided in Table 1. GAPDH was used as housekeeping gene. Activated CD8+ T cells alone was used as reference sample. All samples were run in triplicates. Changes in gene expression were calculated by the ΔΔCT method and depicted as fold change in gene expression compared to control at 24 and 96 h time points as depicted in Fig. 1.

**Table 1:** List of primers used for gene expression

Target gene	Accession number	Sequence	Product size (bp)
GAPDH	NM_001009307.1	Fw: 5′-CCATGTTTGTGATGGGCGTG-3′ Rev: 5′-TGATGGCATGGACTGTGGTC-3′	159
IL-2	NM_001043337.1	Fw: 5′-GCAATTACTGCTGGATTTACGGTTGC-3′ Rev: 5′-AGTCAGCGTTGAGAAGATGCTTTG-3′	358
Granzyme B	XM_006932823.3	Fw: 5′-CCACCCAGACTATAATCCAAAGAA-3′ Rev: 5′-CAGTCAGCTTGGCCTTTTTCA-3′	77
Perforin	NM_001101660.1	Fw: 5′-GGGAGCGCTTTTCCGAAATAG-3′ Rev: 5′-CTGGAAGTTCAT GACCTCCA-3′	380
KLRG-1	XM_023256758	Fw: 5′-AGGAAATGAGCCTGCTTCAA-3′ Rev: 5′-CCGTAAAAGAGCCTCACAGC-3′	194

*Fw* Forward primer, *Rev* Reverse primer

### Statistical analyses

Normal distribution of the data was tested using the Shapiro–Wilk test. A paired t test (normally distributed data) or Mann–Whitney *U* test (non-normally distributed data) was used to determine differences in between two groups in data set. Difference between 3 groups was determined using Kruskal–Wallis test with Dunn’s multiple comparison post-test. A commercially available statistical software was used for all statistical analyses (GraphPad InStat version 3.06 for Windows; GraphPad Prism, San Diego, CA). A *P* value of < 0.05 was considered statistically significant.

## Results

### Feline ASCs induce cell cycle arrest in CD8+ T cells in a contact-dependent manner

We previously described that feline ASCs inhibit lymphocyte proliferation, both with or without cell–cell contact. However, the induction of IFN-γ secretion by CD4+ and CD8+ T cells was enhanced after direct cell contact with ASCs and was mediated through engagement of ICAM-1/LFA-1 ligands [9]. We also determined that the inhibition of lymphocyte proliferation by feline ASCs was due to cell cycle arrest in G0-G1 [3]. Here, we expanded our findings to determine if ASCs induced G0-G1 cell cycle arrest, specifically in CD8+ T cells, and if this arrest was also associated with direct ASC-CD8+ T cell contact.

**Fig. 2:**
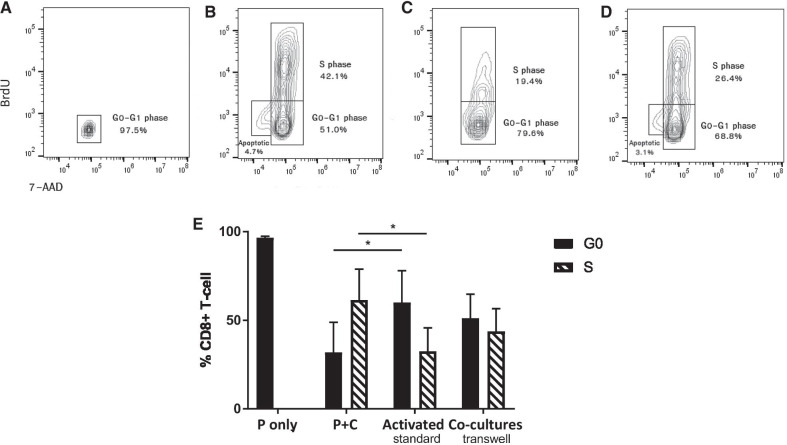
Feline ASCs induce cell cycle arrest in CD8+ T cells in a contact-dependent manner. Representative flow-plot on cell cycle analysis with and without transwell **A** resting CD8+ T cells, **B** mitogen-activated CD8+ T cells, **C** activated CD8+ T cells co-cultured with MSCs in standard condition, **D** activated CD8+ T cells co-cultured with MSCs in transwell, **E** summary of cell cycle analysis with changes percentage of G0-phase and S-phase between activated CD8+ and co-cultures with activation (*n* = 7, *p* value = 0.014 and 0.006, respectively). *P only* PBMCs only, *P+ C* PBMCs with ConA

We found that the induction of cell cycle arrest in CD8+ T cells was more efficient with direct cell–cell contact between feline ASCs and PBMCs (Fig. 2A–D) with a significant decrease of CD8+ T cells in S-phase and increase in G0-G1 phase compared to conA-activated CD8+ T cells alone (*n* = 7, *p* = 0.014 and *p* = 0.006 respectively).

### Feline ASCs downregulate CD8+ T cell surface expression, increase CD25 and CD57 expression, and shift CD8+ T cells toward a terminally differentiated, effector phenotype

**Fig. 3:**
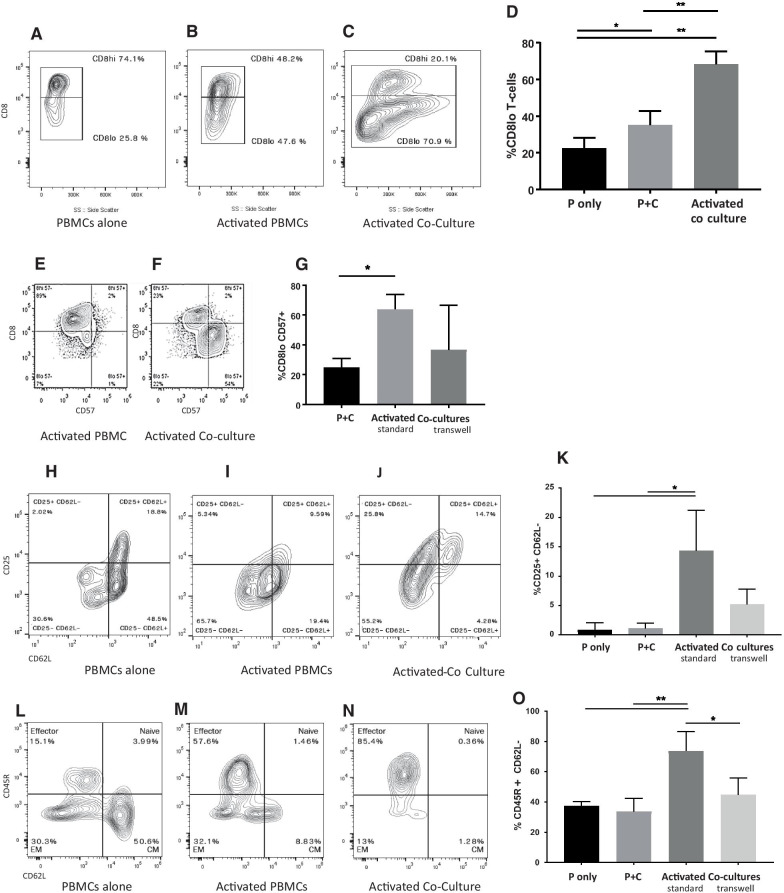
Feline ASCs downregulate CD8+ T cell surface expression, increase CD25 and CD57 expression, and shift CD8+ T cells toward a terminally differentiated, effector phenotype. Representative flow-plot on CD8+ T cell surface expression on **A** non-activated CD8+ T cells **B** activated CD8+ T cells **C** activated CD8+ T cells co-cultured with MSCs. **D** Summary of percentage of CD8lo T cells (*n* = 5, *p*-value = 0.007). CD57 expression on **E** activated CD8+ T cells **F** activated CD8+ T cells co-cultured with MSCs **G** Summary of percentage of ASCs’ induction of CD57+ CD8lo T cells (*n* = 5, *p* value = 0.007). CD25 and CD62L expression on **H** non-activated CD8+ T cells **I** activated CD8+ T cells **J** activated CD8+ T cells co-cultured with MSCs **K** Summary of CD25 and CD62L expression. ASCs increase activation (CD25+) and shedding of L-selectin (CD62L−) on CD8+ T cells (*n* = 5, *p*-value = 0.028). Expression of CD45R and CD62L to distinguish between effector, effector memory, central memory and naïve CD8+ T cells in **L** non-activated CD8+ T cells **M** activated CD8+ T cells **N** activated CD8+ T cells co-cultured with MSCs. **O** Summary of increase effector phenotype (CD45R+ , CD62L-) on CD8+ T cells induced by ASCs (*n* = 5, *p*-value = 0.02)

We demonstrated that feline ASCs downregulate CD8+ receptor on the surface of cytotoxic T cells (CD8^lo^ T cells) in vitro (Fig. 3A–D) when compared between co-cultures between ASCs/PBMCs and activated PBMCs (*p* value = 0.007). Here we further characterized the phenotype of CD8+ T cells after coincubation with ASCs by interrogating the CD8+ T cells with CD25, CD45R, CD57, and CD62L.

These CD8^lo^ T cells primed by feline ASCs significantly upregulated surface CD57 expression compared to activated CD8+ T cells alone (*p* = 0.007) and less so without direct contact (Fig. 3E–G). Feline ASCs also upregulated the expression of CD25+ and decreased the expression of CD62L on CD8+ T cells (Fig. 3H–K, *p* = 0.028), possibly indicative of augmented CD8+ T cell activation with CD62L shedding.

Additionally, after interaction with feline ASCs, the activated CD8+ cells shifted to an effector phenotype (CD45R+, CD62L−) compared to activated cells without ASC interaction (*p* = 0.02) (Fig. 3L–O). This shift to effector cells is less efficient in the absence of direct cell–cell contact, similar to the expression of CD25 and CD57. These findings collectively suggest that CD8+ T cells that have interacted with feline ASCs may become senescent cells or terminally differentiated cytotoxic T cells.

### Feline ASCs do not induce senescence in CD8+ T cells

**Fig. 4:**
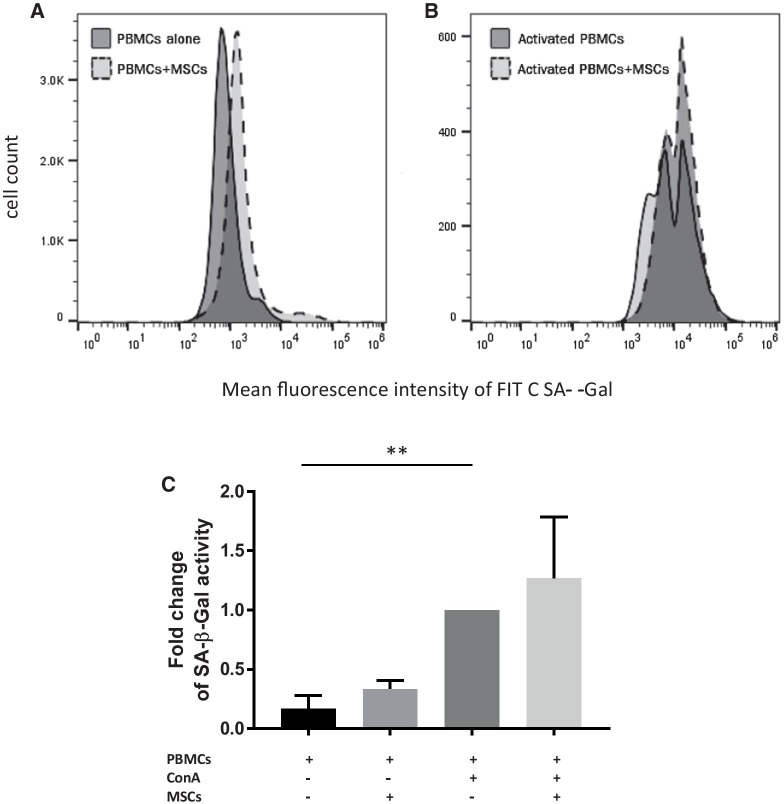
Feline ASCs do not induce senescence in CD8+ T cells. Histogram depicting mean fluorescence intensity for senescence-associated β-galactosidase (SA-β-Gal) positive CD8+ T cells in **A** PBMCs alone and PBMCs in co-culture with MSCs **B** in activated PBMCs and activated PBMCs in co-culture with MSCs **C** Summary of SA-β-Gal activity with no significant increase between activated PBMCs and activated co-cultures and indication of SA-β-Gal activity was mostly from mitogen activation (*n* = 7, *p*-value = 0.007). Fold change calculated based on PBMCs with ConA

To date, our data suggest that ASCs induce poorly proliferative CD8+ T cells that are withdrawn from cell cycle progression, but retain the ability to secrete IFN-γ [9]. These findings could indicate a senescence-associated phenotype. Cellular senescence refers to cells which cease to divide and remain permanently halted in cell cycle progression. We found that mitogen activation induces cell senescence (*p* = 0.007); however, feline ASCs did not significantly induce cellular senescence on CD8+ T cells (Fig. 4A–C).

### Feline ASCs upregulate the expression of granzyme B, IL-2 and KLRG-1

**Fig. 5:**
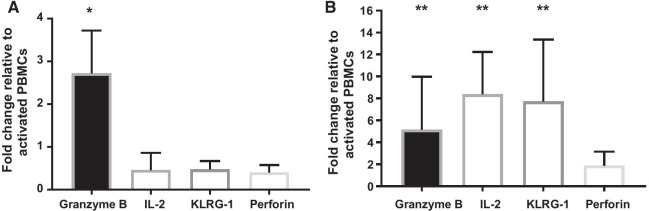
Feline ASCs upregulate the expression of Granzyme B, IL-2 and KLRG-1. **A** upregulation of granzyme B at 24 h from PBMCs in co-culture with ASCs in comparison with activated PBMCs alone (*n* = 6, *p*-value = 0.03) **B** upregulation of granzyme B, IL-2 and KLRG-1 at 96 h from bead-enriched CD8+ T cells in co-culture with ASCs (*n* = 6, *p*-value = 0.002, 0.03 and 0.002 respectively). *IL-2* interleukin 2, *KLRG-1* killer cell lectin-like subfamily G1

PBMCs which have been primed by ASCs significantly upregulated granzyme B gene expression at 24 h compared to activated PBMCs alone (*p* = 0.03, Fig. 5A). There was a concurrent slight downregulation for IL-2, KLRG-1 and perforin expression; however, the changes were not significant. Gene expression at 24 h could not be performed on enriched CD8+ population due to insufficient amount of isolated RNA.

At 96 h, activated PBMCs coincubated with feline ASCs upregulated IL-2 (*p* = 0.002) and KLRG-1 (*p* = 0.03). Granzyme B also remains upregulated at 96 h (*p* = 0.002, Fig. 5B).

### ASC-primed CD8+ T cells acquired enhanced cytotoxicity function on target cells

**Fig. 6:**
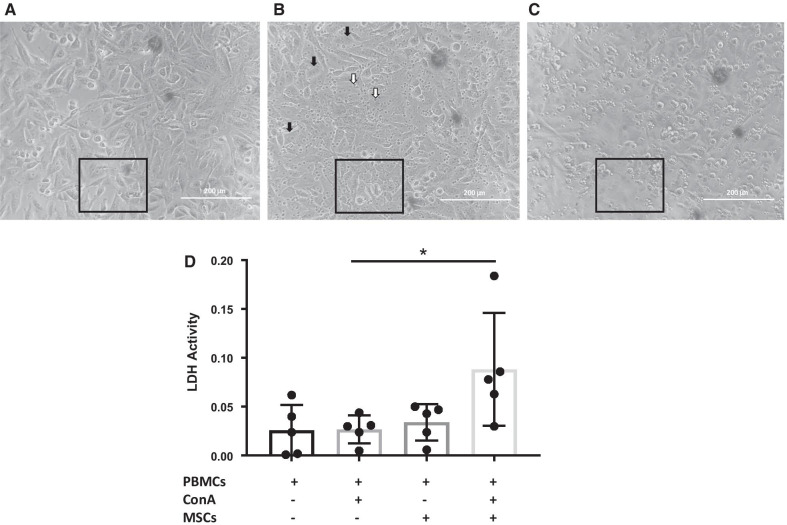
ASC-primed CD8+ T cells acquired enhanced cytotoxicity function on target cells. Inverted microscopic images (400X) of **A** CRFK cells alone in culture **B** CRFK cells in culture with bead-enriched CD8+ T cells from activated PBMCs after 4 h of incubation **C** CRFK cells in culture with bead-enriched CD8+ T cells from co-cultures after 4 h of incubation. Black arrow head indicates CRFK cells; white arrow head indicates enriched CD8+ T cells; boxed areas demonstrate loss of monolayer adherence of CRFK cells **D** Summary of increased LDH activity from lysis of target cells by ASC-primed CD8+ T cells (deducted by spontaneous release of LDH from CRFK cells and enriched CD8+ T cells alone) (*n* = 5, *p*-value = 0.04)

Our data suggest that CD8+ T cells shift to effector cells (CD45R+, CD62L−) with increased Granzyme B expression after incubation with ASCs. We next wanted to determine if the CD8+ T cells were functionally cytotoxic. To test this, we co-incubated ASC primed CD8+ T cells with virus-infected target cells and measured cellular release of lactate dehydrogenase (LDH). Visually, CD8+ T cells that were co-incubated with activated ASCs had strong cytopathic effects on virus-infected target cells (Fig. 6A–C). These visual data were confirmed by measuring LDH release into the supernatant (released upon cell death). We found that CD8+ T cells that had been incubated with feline ASCs had enhanced capacity to lyse viral-infected target cells (*p* = 0.04, Fig. 6D).

## Discussion

Our current work demonstrates for the first time that feline ASCs can alter the phenotypic and functional characteristics of CD8+ T cells toward terminally differentiated effector cells. Although proliferative arrest occurred in feline-ASC primed CD8+ T cells, they remained functionally active and were neither exhausted nor senescent. Activated CD8+ T cells demonstrated early and sustained upregulation of granzyme B after interaction with feline ASCs with later upregulation of IL-2 and KLRG-1, indicative of enhanced cytotoxicity and a shift toward end-stage differentiation.

Feline ASCs have been evaluated for their therapeutic effects in various experimental models and clinical trials relating to inflammatory and immune-dysregulated diseases [1–4, 15, 16]. In feline patients with chronic gingivostomatitis (FCGS), administration of ASCs resulted in decreased CD8+ T cells and the downregulation of CD8 receptor expression, both corresponding to a positive response to ASC therapy [2]. CD8+ T cells play an important role in the pathophysiology of FCGS, especially with a potential underlying cause of viral infection, particularly feline calicivirus (FCV) infection [17].

In this study, we focused on ASC regulation of feline CD8+ T cells as in our clinical trial in cats with FCGS, CD8+ T cells are a clear biomarker associated with infection and phenotype change is associated with disease resolution in some cats. Published in vitro data on CD8+ T cells in mice and people primarily suggest that MSCs dampen T cell proliferation and shift CD8+ T cells toward a suppressive or regulatory phenotype [18–20]; here, we have demonstrated feline ASCs augment CD8+ T cell effector function in vitro.

When CD8+ T cells expand and differentiate during activation, most cells will terminally differentiate into end-stage effectors that have a shortened lifespan and die while a smaller subset of cells differentiates into memory cell precursors [21]. Cytotoxic CD8+ T cells are a crucial component of the adaptive arm of the immune system. One primary role is to combat intracellular pathogens, utilizing granzyme and perforin stored in their cytotoxic granules to kill virally infected cells. Granzyme B, among other members in the granzyme family, has the most potent apoptotic function [22].

Our study showed that feline ASCs induce cell cycle arrest on CD8+ T cells in a contact-dependent manner. Poorly proliferative CD8+ T cells can be the result of a shift to terminal differentiation, functional exhaustion or cellular senescence [23–25]. ASCs do not induce an exhausted phenotype as ASC-primed CD8+ T cells retain their cytokine-secreting ability including IFN-γ [26] and IL-2 expression.

Senescent and terminally differentiated CD8+ T cells have several overlapping cellular features. Replicative cellular senescence is commonly associated with aging and is explained by telomere shortening after cells have undergone numerous replicative cycles [24]. Premature senescence, which is telomere-independent, can be induced by cellular stress, DNA damage or T regulatory cells [27, 28]. Feline ASCs induced CD25 (IL2 receptor) upregulation. IL-2 plays a pivotal role in the survival, clonal expansion and promoting CD8+ T cells toward effector differentiation [29]. Moreover, feline ASCs also induced shedding of L-selectin (CD62L) a lymph node homing receptor, and upregulated CD45R expression, indicative of an effector phenotype. Additionally, cytokine milieu also affects effector cell function and feline ASCs secrete several immunomodulating cytokines and also augment IFN-γ secretion from T-lymphocytes [26], possibly indicative of their drive toward short-lived cytotoxic CD8+ T cells [30].

We also determined that these CD8+ T cells were not senescent through the detection of intracytoplasmic senescence-associated β-galactosidase enzyme in CD8+ T cells. Mitogen stimulation did induce cellular senescence; however, ASCs neither induced senescence nor rescued PBMCs from activation/replication-induced senescence. Given that the CD8+ T cells were not senescent or exhausted and their phenotype was most like effector cells, we confirmed these findings using gene expression and a functional assay.

Killer cell lectin-like receptor subfamily G1 (KLRG-1) is an inhibitory marker expressed on NK cells and T cells, predominantly associated with CD8+ effector cells retaining cytokine production capacity, but limited proliferative ability while approaching the end of T cell differentiation. [31, 32]. Our data showed increased expression of granzyme B and KLRG-1 supportive of CD8+ T cell differentiation. Similar to our findings, in humans, the KLRG-1+ population of CD8+ T cells has increased granzyme B expression [33]. These findings were further confirmed by increased surface expression of CD57, an NK cell and T cell marker most prominently expressed in terminally differentiated effector T cells and mature cytotoxic NK cells [34].

Lastly, we reported enhanced cytotoxic functionality of feline ASC-primed CD8+ T cells through an LDH-release of virally infected target cells. We utilized Crandell-Reese feline kidney (CRFK) cells, an immortalized allogeneic mesenchymal cell line [35], as the cell target to examine the effects of CD8+ T cell-mediated cytotoxicity. Destruction of target cells through TCR-MHC-class I and/or Fas-ligand interaction [36] between activated feline CD8+ T cells and CRFK cells was measured through lactate dehydrogenase (LDH) release. LDH is present in all mammalian cells, and the release in culture supernatant was quantified in comparison with target cells (CRFK) alone and effector cell (enriched CD8+ T cells) alone with maximal lysis control after 4 h of co-incubation. Our cytotoxicity assay indicated an approximately 61% increase of CRFK cell death from feline ASCs primed activated CD8+ T cells. We also noted that the FCV infection on target cells was not necessary for cell lysis (data not shown).

## Conclusion

Our findings collectively suggest that feline ASCs (1) alter CD8+ T cells toward terminally differentiated, proinflammatory (IFNγ, CD25 (IL2 receptor), IL2) effector phenotype (CD8^lo^, CD62L−, CD45R+) with limited proliferative capacity, and (2) enhance their cytotoxic potential through granzyme B upregulation. These data mimic some of our findings in vivo after ASC administration to cats with FCGS, a disease characterized by a CD8+ T cell proinflammatory phenotype. These cytotoxic CD8+ T cells could aid in disease cure in cases caused by an underlying, unresolved viral infection.

## Data Availability

All datasets used and/or analyzed during the current study are available from the corresponding author on reasonable request.

## Abbreviations

ASC: Adipose-derived mesenchymal stem cell
BrdU: 5-Bromo-2'-deoxyuridine
ConA: Concanavalin A
FBS: Fetal bovine serum
IFNγ: Interferon gamma
MSC: Mesenchymal stem cell
PBMC: Peripheral blood monocular cell
DPBS: Dulbecco’s phosphate buffered saline
SPF: Specific pathogen-free
